# Antileishmanial, Toxicity, and Phytochemical Evaluation of Medicinal Plants Collected from Pakistan

**DOI:** 10.1155/2014/384204

**Published:** 2014-05-19

**Authors:** Naseer Ali Shah, Muhammad Rashid Khan, Akhtar Nadhman

**Affiliations:** ^1^Department of Biochemistry, Faculty of Biological Sciences, Quaid-i-Azam University, Islamabad 45320, Pakistan; ^2^Department of Biotechnology, Faculty of Biological Sciences, Quaid-i-Azam University, Islamabad 45320, Pakistan

## Abstract

Leishmaniasis is an important parasitic problem and is in focus for development of new drugs all over the world. Objective of the present study was to evaluate phytochemical, toxicity, and antileishmanial potential of *Jurinea dolomiaea*, *Asparagus gracilis*, *Sida cordata,* and *Stellaria media* collected from different areas of Pakistan. Dry powder of plants was extracted with crude methanol and fractionated with *n*-hexane, chloroform, ethyl acetate, *n*-butanol, and water solvents in escalating polarity order. Qualitative phytochemical analysis of different class of compounds, that is, alkaloids, saponins, terpenoids, anthraquinones, cardiac glycosides, coumarins, phlobatannins, flavonoids, phenolics, and tannins, was tested. Its appearance was observed varying with polarity of solvent used for fractionation. Antileishmanial activity was performed against *Leishmania tropica* KWH23 promastigote. Potent antileishmanial activity was observed for *J. dolomiaea* methanol extract (IC_50_ = 10.9 ± 1.1 **μ**g/mL) in comparison to other plant extracts. However, *J. dolomiaea* “ethyl acetate fraction” was more active (IC_50_ = 5.3 ± 0.2 **μ**g/mL) against *Leishmania tropica* KWH23 among all plant fractions as well as standard Glucantime drug (6.0 ± 0.1 **μ**g/mL). All the plants extract and its derived fraction exhibited toxicity in safety range (LC_50 _ > 100) in brine shrimp toxicity evaluation assay.

## 1. Introduction

Leishmaniasis is an important protozoal disease caused by parasite belonging to genus* Leishmania*. Sand fly is the responsible vector for its transmission. Leishmaniasis is a major health risk and a danger to 350 million populations throughout the world. Approximately, 12 million peoples are currently infected and every year around 1-2 million new cases are appearing [1]. This disease may vary in its presentation; it can be self-healing or fatal. Various infectious types of leishmaniasis can be categorized as (a) cutaneous leishmaniasis; (b) mucocutaneous leishmaniasis; and (c) visceral leishmaniasis. Hereby, about 0.5 and 1.5 million cases of visceral and cutaneous leishmaniasis are reported, respectively. In Pakistan, leishmaniasis was first accounted in northern areas in 1960. In the beginning, it was confined to northern sphere but now it is widely spreading throughout the country [2].

All body parts especially the exposed ones are mainly targeted by cutaneous leishmaniasis. The nascent lesion may give rise to an ulcer. Systemic leishmaniasis affects internal organs of a body, that is, spleen and liver, rare in Pakistan but can prove to be fatal. The multiple ulcers resulting from multiple bites of sand fly are not a rare case in Pakistan. On the other hand, it is rapidly spreading out creating an alarming situation. Cutaneous and visceral leishmaniasis is the major threats to Pakistan, despite of mucocutaneous leishmaniasis which is rarely reported [3].

The treatments currently in use as prime therapy include meglumine antimoniate (Glucantime) in addition to pentavalent antimonials sodium stibogluconate (Pentostam) but they are harmful to some extent, require prolong parenteral administration courses, and have virulent side effects [4, 5]. In some cases, pentamidine as well as amphotericin B are used as second line treatment which may also have lethal affects [6–8]. There is a need to develop novel drugs to counter leishmaniasis due to the existence of various hazards which include high cost of current medicines [9, 10] along with their possible toxic effects [11] and resistance development in parasites [12]. Because of these problems, scientists are in continuous search to find new drugs against these infections. Naturally existing medicinal herbs and plants are considered to be the rich supply of a variety of useful organic substances. As natural compounds are considered good and affluent source of bioactive compounds having antileishmanial ability [13, 14], there is a need for the identification and analysis of various natural products originated from different medicinal plants so that new drugs can be synthesized [15].

From the very beginning, plants having medicinal abilities are being used as a source of treatment against various diseases. According to an estimate, more than 25% of recommended drugs were attained from plants with or without supplementary adjustments [16, 17]. Extensive studies have been done on the agents with antileishmanial characteristics. The extracts or bioactive substances derived from medicinal herbs are possibly the valuable and satisfying source of new therapeutic entities against parasites [14, 18]. A wide variety of such plant species have been computed which enclose potentially effective leishmanicidal compounds [13].

In this study, four important medicinal plants, that is,* Jurinea dolomiaea* Boiss,* Asparagus gracilis* Royle,* Sida cordata* (Burm.f.), and* Stellaria media* (L.), were studied following random plant selection approach of screening.

Plants undertaken in this study are never reported in the literature for the study of antileishmanial activity. So this study was designed to explore its phytochemicals, potential in the treatment of leishmaniasis, and investigate its safety by performing brine shrimp toxicity assay.

## 2. Material and Methods 

### 2.1. Plant Collection

Plants collections were made from the areas shown in 2011. The plants were recognized by their local names and then confirmed by Dr. Mir Ajab Khan, Department of Plant Sciences, Quaid-i-Azam University, Islamabad, and Dr. Zafar, Curator, Herbarium, Quaid-i-Azam University, Islamabad. Voucher specimens with accession nos. 24561(*A. gracilis*), 27813 (*S. media*), 27824 (*S. cordata*), and 27823 (*J. dolomiaea*) were deposited at the Herbarium, Quaid-i-Azam University, Islamabad. Table 1 displays synonym, family, part of the plant, local name, and collection area.

### 2.2. Extract Preparation

After collection, plant samples were shade dried till the complete removal of moisture and samples were made to mesh sized powder by using plant grinder. Powders (5 kg) of each sample were soaked in crude methanol (10 L) for extraction for 72 h. All the samples were processed two times repeating above procedure. For the purpose of filtration, Whatman No. 1 filter was used and methanol was evaporated on a rotary evaporator at 40°C under reduced pressure.

### 2.3. Fractionation

**Figure 1:**
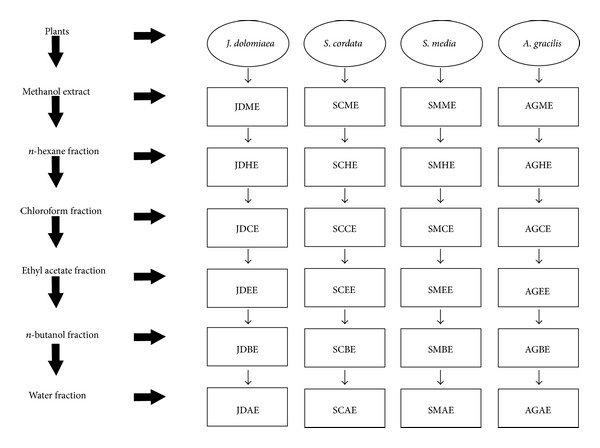
Flow sheet of extraction and fractionation of* J. dolomiaea, S. cordata, S. media,* and* A. gracilis.*

To sort the compounds in the crude extract with increasing polarity, crude extract (6 g) was suspended in distilled water (250 mL) and passed to liquid-liquid partition by using solvents in order of* n*-hexane, chloroform, ethyl acetate, and* n*-butanol. The residue left behind after* n*-butanol fraction was termed as aqueous fraction (Figure 1). Rotary evaporator was used to concentrate the fraction by evaporating the solvent under reduced pressure at 40°C. Fractions were further dried under dark and weighed and stored at 4°C for phytochemical and pharmacological evaluation.

### 2.4. Phytochemical Analysis

Qualitative phytochemical investigation of extracts and fractions for the existence of alkaloids, saponins, terpenoids [19], anthraquinones, cardiac glycosides, coumarins, phlobatannins [20], flavonoids, phenolics, and tannins [21] was performed.

### 2.5. Antileishmanial Activity

Stock solution for antileishmanial assay was prepared by dissolving 5 mg/mL of each plant extract and derived fractions in 1 mL of DMSO (dimethyl sulfoxide). Stock solutions were further diluted serially (2500, 1250, 625, 312.5, 156.3, 78.1, 39.1, and 19.5 *μ*g/mL) using DMSO to obtain the concentration from 333.3 to 1.3 *μ*g/mL in the wells. Samples were filtered by using a 0.45 *μ*m syringe filter.* Leishmania tropica* KWH23 was previously isolated from a patient in Peshawar, Pakistan, and was characterized (data not shown). The promastigotes form of the Leishmania were grown in M199 medium with 10% fetal calf serum (FCS), HEPES buffer, streptomycin, and penicillin. Log phase promastigotes at 1 × 10^6^/100 *μ*L were used for the entire assay. About 90 *μ*L of 199 media, 50 *μ*L of* Leishmania tropica* KWH23 log phase culture, and 10 *μ*L of each plant dilution were dispensed to different wells of microtitter plate. Here, DMSO was used as a negative control while Glucantime as positive control. Afterwards, microtitter plate was incubated at 24°C for 72 h. After incubation, about 15 *μ*L of each dilution was pipetted on a neubauer counting chamber and was counted under a microscope.

### 2.6. Toxicity Studies

To estimate the toxicity of the plant extracts and fractions, brine shrimp lethality assay was used. For the hatching of brine-shrimps (*Artemia salina*) eggs, at an ambient temperature of 23 ± 1°C artificial sea water (3.8 g sea salt/L) was used [22]. Three different concentrations of each extract (2500, 500, and 50 *μ*g/mL) were made, taken from 10 mg/mL stock solution in methanol. Methanol was evaporated before transferring shrimps to the vials. After 24 h, the hatched shrimps were shifted to the vials filled with 5 mL of artificial sea water (10 shrimps per vial) along with samples. The number of the shrimps that survived the sample environment was counted after 24 h. Tricaine methanesulfonate was used as standard.

### 2.7. Statistical Analysis

Graph Pad Prism software version 5.0 (2007) was used to calculate LC_50_ and IC_50_ values of each sample for brine shrimp toxicity assay and antileishmanial activity. Nonlinear regression test was used to determine* R* ^2^, LC_50_, and IC_50_ values.

## 3. Results

### 3.1. Qualitative Phytochemistry

In qualitative phytochemical investigation,* J. dolomiaea* methanol extract and JDCE fraction showed the presence of all the classes of phytochemicals determined in this study except phlobatannins that was not recorded in JDCE. However, flavonoids, phenolics, alkaloids, and phlobatannins were not observed in JDHE whereas JDEE showed absence of alkaloids and tannins. In JDBE alkaloids, phlobatannins and saponins were lacking while JDAE showed the presence of only saponins, cardiac glycosides, and phenolics.

Phytochemicals appearances were altered in different fractions with a change of nature of fraction solvent. Anthraquinones and phlobatannins were absent in all fractions of* A. gracilis *as well as extract. AGHE, AGCE, and AGAE expressed the presence of only tannins, saponins, and cardiac glycosides. AGEE illustrated absence of tannins and saponins. Flavonoids, phenolics, saponins, and alkaloids were absent in AGBE.

In case of* S. cordata,* SCME, SCCE, and SCEE showed the presence of all kinds of chemical classes. Alkaloids, anthraquinones, phlobatannins, cardiac glycosides, and coumarins were not reported in the SCHE. SCBE fraction did not possess the anthraquinones, phlobatannins, and coumarins while in SCAE alkaloids, anthraquinones, phlobatannins, tannins, and coumarins were absent.

Anthraquinones and phlobatannins were absent in crude methanol extract of* S. media*. Alkaloids, anthraquinones, phlobatannins, and cardiac glycosides were not found in SMCE and SMEE. Only saponins, cardiac glycosides, flavonoids, and phenolics showed their presence. Terpenoids, saponins, cardiac glycosides, flavonoids, and phenolics were observed in SMHE and SMAE.

### 3.2. Antileishmanial Activity

Table 2 displays IC_50_ values of plants studied for antileishmanial activity.* Jurinea dolomiaea *JDEE fraction exhibited the best activity in terms of IC_50_ value (5.3 ± 0.2 *μ*g/mL) comparably low than standard drug Glucantime (5.6 ± 0.25) against* Leishmania tropica* promastigotes. Highest IC_50_ was expressed by JDCE. IC_50_ values of JDME, JDEE, JDHE, and JDAE fall in a range with minor differences. Regression square (*R* ^2^) value ranged 0.81–0.9.

In case of* A. gracilis,* AGAE showed the lowest IC_50_ (12.6 ± 1.5 *μ*g/mL) while the highest IC_50_ was displayed by AGHE (36.6 *μ*g/mL). AGME, AGCE, AGEE, and AGBE demonstrated IC_50_ values of 33.9 ± 1.5, 28.3 ± 3.5, 13.5 ± 0.7, and 18.9 ± 1.2 *μ*g/mL, respectively. Regression square (*R* ^2^) value ranged 0.79–0.98.

SCHE exhibited the lowest IC_50_ (9.2 *μ*g/mL) in* S. cordata* samples while the highest by SCAE (259.1 ± 12.5 *μ*g/mL). SCEE, SCCE, SCME, and SCBE showed IC_50_ values of 56.8 ± 5.5, 125.5 ± 4.5, 41.8 ± 2.1, and 228.5 ± 10.2 *μ*g/mL, respectively. Regression square (*R* ^2^) value ranged 0.74–0.98.

Methanol extract of* S. media* demonstrated IC_50_ value of 185.9 ± 7.5 *μ*g/mL. However, SMEE displayed the lowest IC_50_ value of 36.4 ± 2.5 *μ*g/mL followed by SMBE, SMCE, SMHE, and SMAE. Regression square (*R* ^2^) value ranged 0.85–0.98.

### 3.3. Brine Shrimp Toxicity

Brine shrimp toxicity is an easy and economical* in vitro *assay to determine toxicity and safety of crude extract. Brine shrimp* in vitro* assay was performed to evaluate the safety assessment extracts and its derived fractions. Table 2 displays toxicity data of plants. JDME extract showed LC_50_ of 733.0 ± 15.1 *μ*g/mL. In derived fractions, LC_50_ ranged from 569.5 ± 7.4 to 1593 ± 20.2 *μ*g/mL. Lowest LC_50_ was shown by JDEE while higher by JDAE. Regression* R* ^2^ ranged 0.92–1.0.

AGME showed LC_50_ of 321.5 ± 11.5 *μ*g/mL. In derived fractions, LC_50_ ranged from 211.9 ± 3.7 to 588.63 ± 7.2 *μ*g/mL. The lowest LC_50_ was displayed by AGEE while higher LC_50_ value was exhibited by AGBE. Regression* R* ^2^ ranged 0.71–0.97.

SCME extract of* S. cordata* showed LC_50_ of 125.7 ± 1.5 *μ*g/mL. However, LC_50_ value of its derived fractions expressed higher values and ranged from 211.9 ± 2.9 to 882.4 ± 6.3 *μ*g/mL. The lowest LC_50_ was shown by SCAE while the highest by SCBE. Regression square ranged 0.75–0.99.

In case of* S. media,* SMME extract showed LC_50_ value of 436.7 *μ*g/mL whereas its derived fractions exhibited comparatively higher LC_50_ values and ranged from 660.7 ± 2.5 to 789.3 ± 2.7 *μ*g/mL. The lowest LC_50_ was shown by SMAE while the highest by SMEE. Regression square ranged 0.85–0.99.

## 4. Discussion

Phytochemical screening is the first step in herbal medicine research to identify bioactive and novel lead compounds. Plant material consists of many different kinds of natural products with nature of different polarities leading to a different mode of solubility [23, 24].

In the present study, extraction with crude methanol was obtained by soaking for 4-5 days with frequent shaking in the ratio of 1 kg powder : 2 liter of crude methanol. Sequential fractionation with escalating polarity of solvent,* n*-hexane, chloroform, ethyl acetate, and* n*-butanol was followed in this experiment. In the end, residue portion was termed as aqueous fraction.

In qualitative analysis, crude methanol extract illustrated maximum number of different classes of compounds for all the plants under present study. Similar observations were reported by Saeed et al. [25] and Rashid et al. [26] in their studies on* Torilis leptophylla* and* Fagonia olivieri, *respectively. Preliminary qualitative screening which ascertains these plants may have potential bioactive components. The present study reports that* J. dolomiaea, A. gracilis,* and* S. cordata* are potential source of bioactive compounds in term of flavonoids, phlobatannins, phenolics, alkaloids, anthraquinones, saponins, coumarins, tannins, terpenoids, and cardiac glycosides.* Stellaria media* also showed all classes except anthraquinones and phlobatannins. The presence of such bioactive components explains the use of these plants in the folk medicine.

Leishmaniasis is nowadays an important parasitic problem in the world. There is a need to identify new drugs against the parasite causing leishmaniasis. Antileishmanial activity was performed to expose potential in inhibiting and retarding multiplication of leishmanial parasite.

Different fractions of methanol extract of the plants used in this study indicated that JDEE of* J. dolomiaea*, SMEE of* S. media* exhibited the lowest IC_50_ among the different fractions. This activity may be due to the high content of flavonoids and phenolics. García et al. [27] have reported the flavanols constituents of* Pluchea carolinensis* as antileishmanial agents. Some other studies showed the presence of phytochemicals possessing antileishmanial activity such as flavones [28] and alkaloids [29]. Kolodziej et al. [30] verified polyphenols for their antiparasitic activity. Marín et al. [31] analyzed couple of flavonoids from* Consolida oliveriana*. Antileishmanial activity versus promastigote and amastigote type documented pertaining to kaempferol, quercetin, and trifolin along with their O-acetyl derivatives and acetyl hyperoside as well as octa-O-acetyl hyperoside. These compounds were observed to be noncytotoxic and with efficacy equal or lower than the reference drugs, that is, Pentostam and Glucantime. SCHE presented the lowest IC_50_ among* S. cordata* and AGAE for* A. gracilis*, proposing some other bioactive phytochemical other than flavonoids and phenolics. Thus, there is a need of further study to investigate the active compounds responsible for antileishmanial activity and its* in vivo* application to sought out its appropriate concentration against* Leishmania* in infected experimental organisms. It would be helpful in designing new medicines which are biologically more active and cost-effective and having minimum side effects.

Most of the pharmacologically active compounds are obtained from the natural sources. Because of the traditional use, most of them are safely utilized nowadays. But when we move towards new discoveries from the medicinal plants, the toxicity profile of the respective plant must also be checked in order to enhance safety [32]. Pharmacological evaluation of plants provides a good source for the development of novel and safe medicinal plants. The brine shrimp toxicity is considered an inexpensive, rapid, convenient, and reliable assay for determination of toxicity of a pure compound or medicinal plant extracts or fractions [33]. In the present study, all the plant extracts and their derived fractions showed LC_50_ values in safety range following the statement of Peteros and Uy [34], placing crude extract in nontoxic having LC_50_ > 100 *μ*g/mL for brine shrimp.

Krishnaraju et al. [35] investigated 120 plants aqueous extract toxicity by using brine shrimp lethality study.* Pistacia lentiscus* showed lower LC_50_ of 2.5 *μ*g/mL.* Boswellia serrata*,* Aristolochia indica*,* Garcinia cambogia*,* Ginkgo biloba*, and* Semecarpus anacardium* showed significant toxicity with LC_50_ of 18, 13, 22, 21, and 29.5 *μ*g/mL, respectively. Additionally, Krishnaraju et al. [36] reported brine shrimp toxicity of aqueous extracts of 118 Indian medicinal plants and discovered eleven out of the 118 extracts with critical harmfulness to the brine shrimp (LC_50_ < 60 *μ*g/mL).* Polygonum cuspidatum* and* Syzygium cumini* extracts have shown strong action with LC_50_ of 13.5 and 20 *μ*g/mL, respectively.

In the present study, brine shrimp toxicity study also supports the acute toxicity study where fractions used for the* in vivo* studies in animal models were found safe with high doses.

## 5. Conclusion

The results depict that the plants described above have highly potent compounds involving in antileishmanial activity and they can be used as an effective mean against* Leishmania*. Therefore, further studies are required for the isolation of these effective compounds and* in vivo* implementation.

## Figures and Tables

**Table 1:** Plant synonym, family, part, local name, and collection area of plants under study.

Plant	Synonym	Family	Part	Local Name	Collection Area
*A. gracilis *	*Asparagus capitatus subsp. gracilis *	Asparagaceae	Aerial	Sha gandal	Islamabad
*S. media *	Alsine* media L *	Caryophyllaceae	Whole plant	Gander	Islamabad
*S. cordata *	*Sida veronicaefolia *	Malvaceae	Whole plant	Simak	Islamabad
*J. dolomiaea *	*Jurinea macrocephala *Royle	Asteraceae	Roots	Nazar zela	Kohistan (KPK)

**Table 2:** Antileishmanial and toxicity activity of *J. dolomiaea*, *A. gracilis*, *S. cordata,* and *S. media* methanol extract and its derived fractions.

Sample	Antileishmanial		Toxicity	
	*R* ^2^	IC_50_ (*μ*g/mL)	*R* ^2^	LC_50_ (*μ*g/mL)
*J. dolomiaea *				
JDME	0.84	10.9 ± 1.1	1.00	733.0 ± 15.1
JDHE	0.95	7.2 ± 0.5	0.98	982.5 ± 12.3
JDCE	0.81	47.7 ± 2.3	0.98	834.5 ± 8.5
JDEE	0.98	5.3 ± 0.2	0.98	569.5 ± 7.4
JDBE	0.97	21.8 ± 2.4	0.95	958.3 ± 10.8
JDAE	0.97	6.0 ± 0.1	0.92	1593 ± 20.2
*A. gracilis *				
AGME	0.82	33.9 ± 1.5	0.71	321.5 ± 11.5
AGHE	0.79	36.6 ± 1.4	0.97	280.6 ± 4.3
AGCE	0.94	28.3 ± 3.5	0.86	383.5 ± 9.5
AGEE	0.92	13.5 ± 0.7	0.94	211.9 ± 3.7
AGBE	0.95	18.9 ± 1.2	0.84	588.6 ± 7.2
AGAE	0.98	12.6 ± 1.5	0.89	460 ± 12.5
*S. cordata *				
SCME	0.90	41.8 ± 2.1	0.99	125.7 ± 1.5
SCHE	0.90	9.2 ± 0.7	0.75	879.5 ± 1.3
SCCE	0.89	125.5 ± 4.5	0.84	802.8 ± 4.7
SCEE	0.74	56.8 ± 5.5	0.94	309.9 ± 3.3
SCBE	0.98	228.5 ± 10.2	0.92	882.4 ± 6.3
SCAE	0.92	259.1 ± 12.5	0.94	211.9 ± 2.9
*S. media *				
SMME	0.95	185.9 ± 7.5	0.99	436.7 ± 8.2
SMHE	0.99	170.4 ± 5.5	0.88	542.5 ± 10.4
SMCE	0.83	155.5 ± 3.8	0.98	600.0 ± 6.5
SMEE	0.85	36.4 ± 2.5	0.85	789.3 ± 2.7
SMBE	0.93	49.5 ± 3.5	0.95	760.2 ± 13.6
SMAE	0.98	184.8 ± 5.8	0.96	660.7 ± 2.5
Glucantime	0.99	5.6 ± 0.25	—	—
Tricaine methanesulfonate	—	—	0.93	4.3 ± 0.15

—: represents activity not performed.
